# Finnish UNESCO school educators’ understanding of global citizenship education: Analysis through typologies, ecosocial understanding, and human rights

**DOI:** 10.1007/s11125-021-09597-z

**Published:** 2022-04-11

**Authors:** Tuija Kasa, Laura Karilainen, Antti Rajala, Hannele Cantell, Arto Kallioniemi

**Affiliations:** 1 University of Helsinki, Siltavuorenpenger 5A, P.O. Box 9, 00014 Helsinki, Finland; 2 University of Oulu, PL 8000, 90014 Oulun yliopisto, Oulu, Finland

**Keywords:** Global citizenship education, UNESCO ASPnet schools, Mixed methods, Curriculum, Ecosocial understanding, Human rights

## Abstract

This article sheds light on the unexplored field of UNESCO schools in Finland, and the results clarify the relationships between curricula, international commitments, and the understanding of educators in the educational field. It examines how teachers and principals of UNESCO’s Associated Schools Network (ASPnet) in Finland describe their understanding of the role of global citizenship education (GCE). It draws on the typology proposed by Oxley and Morris in which forms of global education are divided into cosmopolitan types and—more critically—advocacy types and subtypes. The article also draws on concepts connected to GCE in the Finnish curricula (namely, ecosocial understanding and human rights). Findings indicate that educators perceived equality, democracy, and ecological sustainability as part of UNESCO schools and their own work. On the other hand, the need for increasing student-centered approaches was noted, racism was perceived as a difficult topic, and active deconstruction of inequalities was less referenced. When analyzing the results through typologies of global citizenship, the critical, spiritual, and economic aspects of GCE received less attention.

The importance of global citizenship education (GCE) for school curricula is becoming widely recognized internationally, and GCE is being actively promoted by several international organizations, such as UNESCO, OECD, and the World Bank (Bourn, 2020). GCE is also centrally connected to the UN’s Sustainable Development Goals (SDGs) and the emphases and themes of the related global 2030 Agenda (Lehtomäki & Rajala, 2020). GCE has gained in importance in the face of today’s societal tendencies, such as youth and children’s climate activism (Fridays for Future), hate crimes (Rauta, 2017), racism and racialization (FRA, 2017), the questioning of international organizations, and the rise of autocratization (Lührmann & Lindberg, 2019). Furthermore, the wide dissemination of disinformation highlights the importance of school systems’ educating people to understand complexity, critical thinking, and global knowledge in an increasingly interrelated world (Gordon, 2018; Mason et al., 2018).

GCE can be interpreted and theorized in a range of different, contested, and even mutually contradictory ways, reflecting underlying values, worldviews, and ideologies (Oxley & Morris, 2013; Pashby et al., 2020). There are distinct regional emphases in how the role of GCE is understood. Goren and Yemini’s (2017) review highlighted variations in approaches to GCE in research, suggesting that in Europe, global education (GE) curricula have emphasized immigration, war, and adjustment to multiculturalism. Citing a single study (Andreotti et al., 2015), Goren and Yemini maintain that Finnish GE is conceived mostly in terms of learning about the other but without much critical perspective. A review by Lehtomäki and Rajala (2020) that included Finnish studies more broadly showed a more varied picture of Finnish GE research. Despite increasing interest in GCE and new studies on how it is approached in research in various geographical locations, little is known about how educators in different parts of the world understand the role of GCE and how they implement it in their educational practice (Bosio, 2020).

Our theoretical position is informed by the emerging critical scholarship in GCE (e.g., Andreotti, 2006; Pashby et al., 2020; Yemini et al., 2019), which has emphasized the need for a critical stance on GCE in both theory and practice. One aspect of our research is exploring how this paradigm shift can be read in the data collected from responses to questionnaires by UNESCO school staff.

In Finland, GE and related educational approaches such as democracy education, human rights education (HRE), citizenship education, multicultural education, and environmental education have relatively strong normative support in policy documents (for example, in the national curricula) (FNAE, 2014; 2018, 2019). In Finland, teachers are obliged to follow these curricula. However, teachers’ autonomy and freedom to interpret the curricula are very broad, and there are no inspection systems or regular national testing in schools (Niemi et al., 2012). In 2014, during the latest major curricula reform at the national level, GCE was strengthened. However, although GCE is featured prominently in the latest National Core Curriculum for Basic Education, there are no indications of critical forms of GCE, such as critiques of charity-based perspectives, the complicity of Western countries in the worsening of global problems, or the root causes of globally unequal distribution of power and wealth (Nasib, 2020). In this study, we chose to analyze educators’ understanding of the role of GCE in general, as well as their understanding of two specific features of the Finnish curricula: ecosocial understanding and human rights. Ecosocial understanding was included in the curricula (FNAE, 2014) for the first time, while human rights was strengthened. Responsibility in relation to international and domestic human rights law has been specifically emphasized (FNAE, 2014, 2018, 2019). In addition to paying attention to the critical aspects, we also seek to study how educators have understood and integrated these new contents and perspectives into their work and how these reforms emerge in their understanding of GCE.

Although human rights are an explicit part of curricula at all levels, HRE research has been scant in Finland (Lehtomäki & Rajala, 2020), and teachers have seen HRE as “obvious yet alien” (Matilainen, 2011), HRE has been detached from its judicial context, and its realization is unsystematic in teacher education (HRC, 2014; Kasa et al., 2021). We also acknowledge the potentially conflicting conceptualizations of human rights and ecosocial understanding: Ecosocial understanding criticizes the notion of prioritizing humans and emphasizes understanding them as embedded in ecological systems (Salonen & Konkka, 2015). Human rights have been criticized for being Western-centric, emphasizing individuality (Santos, 2002), as well as for being “declarationist” and human-centric (Zembylas & Keet, 2019). Teachers have been worried about teaching about rights instead of duties (Cassidy et al., 2014). However, in terms of educational praxis, we do not want to strengthen these divisions; In educational contexts, human rights should be understood as part of social, moral, economic, cultural, and ecological sustainabilities. The judicial aspect of HRE—instead of being reduced to declarationism—should be seen as an important aspect of a democratic society. Another important feature of critical HRE should be the critique of power relations. Hence, the mere notion of human rights as simplistic “moral” conventionalism (e.g., Andreotti, 2006) is problematic, although in practice, it may have these kinds of forms.

We conducted our research in the context of UNESCO schools in Finland. Finnish UNESCO schools are an unexplored field, and the previous curricula reforms provide an interesting ground for researching the understanding of GCE among UNESCO school educators. We have chosen to study UNESCO schools since they focus explicitly on GCE, and in relation to it, may thus represent the more “active” schools. The aim of this study is to outline general dispositions that arise from wide conceptualizations of GCE.

Our research questions are:

1. How do Finnish UNESCO school educators understand the role of global citizenship education (GCE), and how do they implement GCE in their schools/classrooms?
2. What values, emphases, and typologies of GCE can be identified in Finnish UNESCO school educators’ descriptions of how they understand the role of GCE and how they implement it in their schools?
3. How do Finnish UNESCO school educators understand the roles of ecosocial understanding and human rights, and how do they implement them in their schools and classrooms?

In this article, we briefly introduce our way of conceptualizing GCE in the Finnish context, report on our quantitative and qualitative analysis of answers to the half-structured questionnaire (n=81), and discuss the relevance of our findings by comparing them to theoretical and governmental articulations of GCE.

## Conceptions of global citizenship education

The Maastricht Declaration (2002) of the Council of Europe defines GE in terms of five dimensions: Development Education (DE), Human Rights Education (HRE), Education for Sustainability, Education for Peace and Conflict Prevention, and Intercultural Education. The modern concept of sustainable development is derived from the Brundtland Report (World Commission on Environment and Development, 1987), which defined it as “development that meets the needs of the present without compromising the ability of future generations to meet their own needs”. The concept of sustainability is often seen through three interconnected domains: ecological/environmental, economic, and social. In addition, a fourth—cultural—is often seen as its own domain (Hawkes, 2001). The concept has faced some criticism, and currently, its contents are seen as having a much wider scope like in United Nations SDGs (UN, 2015, 2019). The Expert Panel on Sustainable Development (2016) has described the objectives for sustainable development in Finnish society: equal prospects for well-being, a participatory society for all, sustainable practices at work, a sustainable, carbon-neutral society, sustainable local communities, a resource-wise economy, lifestyles respectful of the carrying capacity of nature, and decision-making that is respectful of nature. Today in Finland, GE is connected centrally to the United Nations SDGs and to the content and emphases of the Agenda 2030 Program designed to promote those goals (Lehtomäki & Rajala, 2020).

There are many ways to conceptualize GCE. Its forms can be categorized, for example, according to how much they question or reproduce existing power relations and structures (Andreotti, 2006; Conolly et al., 2019). GCE has also been categorized in terms of its underlying worldviews and orientations (e.g., neoliberal, liberal, critical) (Pashby et al., 2020). In this article, we draw on the typology proposed by Oxley and Morris (2013), which divides forms of GCE into cosmopolitan and—more critically—advocacy types. Cosmopolitan types include political, moral, economic, and cultural global citizenship, and advocacy types include critical, environmental, and spiritual global citizenship (p. 306). Lehtomäki and Rajala (2020) reviewed Finnish research on GE and found that it includes both cosmopolitan and advocacy types, with a trend toward more critical, advocacy-based approaches. The same development can be seen in the international GE literature (Cole, 2017; Conolly et al., 2019). Although we acknowledge the limitations of this typology and do not regard these concepts as fundamental, we nevertheless see it as a relevant heuristic for distinguishing levels of GCE in our research.

UNESCO defines global citizenship education (GCED) through three domains of learning: cognitive, socio-emotional, and behavioral. In the cognitive domain, learners develop skills for understanding issues not only on a national but also on a global as well as national level; they are also able to understand the underlying interdependencies. The socio-emotional domain enhances learners’ sense of belonging to a global community, emphasizes shared values, and develops feelings of empathy and an understanding of diversity. The behavioral domain encourages learners to act responsibly in order to create a more peaceful and sustainable world and fosters active citizenship (UNESCO, 2020).

## GCE in the Finnish context

Finland’s latest national core curriculum was introduced in 2014 and implemented in 2016 (FNAE, 2014). In the chapter of the curriculum focusing on the task of basic education and its general goals (FNAE, 2014), GCE is mentioned as its own entity. GCE is seen as an opportunity to emphasize justice and sustainable development in education, using the UN’s framework for development goals. Schools are also encouraged to collaborate with other schools and organizations developing school activities and education abroad. In this section, we present the foci of our research: ecosocial understanding and human rights in the curricula.

### Ecosocial understanding

In the Core Curriculum for Basic Education (FNAE, 2014), the so-called ecosocial approach is seen as a basic value of education. Salonen and Konkka (2015) describe it as a holistic and multidisciplinary approach to well-being that will facilitate the analysis and management of the world’s complexity from a socio-ecological perspective. Ecosocial education is based on the reality that without a well-functioning biosphere, there can be no society, and without society there can be no societal functions, including an economy. Fundamentally speaking, all serious problems in the Anthropocene era, such as climate change, are global and have social and environmental bases. This is the reason why ecosocial understanding must be in the background of all education (FNAE, 2014).

### Human rights

Human rights are part of a values basis, binding obligations for schooling and content in teaching as general and specific parts of all Finnish national curricula (FNAE, 2014, 2018, 2019). Human rights are present as specific content in various subjects, such as secular ethics, religion, social studies, languages, and others (FNAE, 2014, 2019). A good example can be taken from social studies in grades 4–6, which is compulsory for all pupils: “The pupils are guided to act in a pluralistic society that understands diversity and respects human rights and equality in accordance with values and principles of democracy” (FNAE, 2014).

## Methodology

We conducted a survey to gather research data from UNESCO ASPnet schools in Finland and applied qualitative and quantitative analysis to the data collected.

ASPnet is the largest school network in the world, with approximately 11,700 educational institutions globally and 60 in Finland (FNAE, 2020). The network was established in 1953 to promote UNESCO’s principles in schools and to promote peace in the global sphere. ASPnet works toward realizing Target 4.7 in GCED and Education for Sustainable Development of the UN’s SDGs (UNESCO Associated Schools, 2020). SDG Target 4.7. states that “all learners acquire the knowledge and skills needed to promote sustainable development, including, among others, through education for sustainable development and sustainable lifestyles, human rights, gender equality, promotion of a culture of peace and nonviolence, global citizenship and appreciation of cultural diversity and of culture’s contribution to sustainable development”. Previously, UNESCO schools’ reports have reinforced the impression that UNESCO activities have been reported mainly as a form of internationalization or “traveling” (in the sense of getting acquainted with different cultures). In recent reports, however, UNESCO activities have been connected to UNESCO’s broader goals (e.g., democracy, human rights, sustainability) (personal email communication, January 11–12, 2021). Needless to say, superficially linking “traveling” to UNESCO activities is insufficient for understanding GCE.

## Research participants

The data (n=81) consist of survey responses of 18 principals or vice principals, 8 elementary school teachers, 50 subject teachers, 2 vocational teachers, 2 special education teachers, and one principal/subject teacher. Most of the subject teachers taught language or history. Other subjects mentioned were art, physical education, math, chemistry, physics, biology, geography, social studies, psychology, philosophy, secular ethics, religion, IT, and student counseling.

The educational institutions reported consisted of elementary schools (12.3%), upper comprehensive schools (19.8%), upper secondary schools (60.5%), and vocational schools (7.4%). Geographically, the institutions varied in Finland in the following order: Northern Finland was most represented with 32.1% of answers, Southern Finland 27.2%, Western Finland 22.2%, Eastern Finland 11.1%, and Central Finland 7.4%. We also surveyed whether the teachers are mainly responsible, responsible only to some extent, or not responsible at all with regard to their roles in UNESCO activities in schools. The data are interesting in that the responses (n=78) were distributed rather evenly: 38.5% of the teachers were mainly responsible, 35.9% were not responsible at all, and 25.6% were to some extent responsible for UNESCO activities.

### Questionnaire and data collection

A half-structured electronic questionnaire included statements, Likert-scale options (1 = not at all, 2 = to a small extent, 3 = to some extent, 4 = to a moderate extent, 5 = to a large extent) and open-ended questions to obtain qualitative data. The questions were constructed to reflect the general concepts of sustainability (environmental, social, ecological, and economic), the Maastricht Declaration (2002), and UNESCO’s GCE definition. To go deeper, we also included statements connected to typologies in academic literature (Andreotti, 2006; Oxley & Morris, 2013).

The data were collected in spring 2020 with the assistance of FNAE, which coordinates the national UNESCO ASPnet schools in Finland. The questionnaire was sent via email to principals of UNESCO schools (60), who were each asked to distribute it to three teachers: one who actively participates and is responsible for UNESCO activities and two who do not have an active role. The study was also announced at the annual meeting of the UNESCO schools. However, the number of respondents remained relatively low. This may be because the survey was circulated in schools during the Covid-19 pandemic. We do not know how principals handled the survey, and we could not check if the questionnaire was distributed in the intended manner.

### Analysis methods

We used both qualitative and quantitative analysis. The open-ended qualitative data produced by the half-structured questionnaire were analyzed by means of content analysis; the data are not representative. The quantitative analyses were descriptive, including percentages, frequencies, and standard deviation. These were analyzed by the Statistics Package for Social Sciences (SPSS) program. Since we could not find an appropriate questionnaire for our purpose in the earlier research, we developed our own; however, a limitation of our study is that this questionnaire was not tested for validity.

We draw from content analysis, which is a systematic set of procedures for rigorous analysis, examination, and verification of the contents of the written data (Tuomi & Sarajärvi, 2018). We used data reduction as a key element of our analysis, striving to respect the *quality* of the qualitative data (Cohen et al., 2007); categories and themes were derived from theoretical constructs or areas of interest devised in advance of the analysis—in this case, different concepts and levels of GCE.

The content analysis and construction of themes (Tuomi & Sarajärvi, 2018, chapter 4.1) were carried out at several levels. First, the material was read through carefully to obtain a general understanding of the responses. Second, coding and themes were established systematically to see the frequencies. After this, representative quotes were chosen for this article. The reproducibility and reliability of the coding (Weber, 1990) were controlled by discussions in the research group on the interpretation of the data and the results. Our analysis paid special attention to the emergence of sustainability issues connected to ecosocial understanding across the data and open-ended question 16 (a problematic topic of GCE). By problematic topic, we mean that it raised the kinds of issues or themes that cause uncertainty for teachers based on their subjective experience and the kinds of difficulties they experience in relation to GCE. This caught our attention because of its societal significance, and the answers gave valuable insights on needed emphasis and suggestions for further development of GCE in the future. We focused on key issues that were essential for researching and understanding GCE.

The limitations of the methodology include interpretation biases intrinsic to qualitative data analysis and theory-oriented analysis with regard to categorizing educators’ experiences through researcher-derived theory (Cohen et al., 2007). The interpretation or generalizability of the qualitative and quantitative results is limited since the amount of data is not representative of UNESCO schools in Finland or of specific teachers. However, our data give an overview of what types of issues emerge and what emphases regarding GCE exist in UNESCO educators’ responses.

## Results

First, we present our results as describing general dispositions (UNESCO school activities, educators’ understanding of the role of GCE, and described obstacles). Second, we present the curricula emphases of ecosocial understanding and human rights, which were the special focus of our analysis.

### The role of GCE and UNESCO activities in schools

The first questions concerned UNESCO activities and the meaning of the network for its members. Approximately 42% of the respondents reported having UNESCO activities at their school on a monthly basis, 25% on a semester basis, 18% on a weekly basis and 15% on daily basis.

To better understand how GCED (CGE) is approached in schools, we compiled a list of topics drawn from the different conceptualizations of CGE (see Table 1) and asked to what extent they are handled at schools. Regarding educators’ own activities in teaching and school management, the topics dealt with most frequently were equality in general, gender equality, sustainable development, getting acquainted with different countries and cultures, and democracy, whereas topics related to racialization, development, cooperation, and dialogue on religion and worldviews clearly received less attention. This indicates that moral and social aspects of GCE receive more attention than spiritual, economic, or critical ones (Oxley & Morris, 2013). The view of GCE activities at the broader school level did not significantly differ from the educators’ own activities. However, sustainable development and climate change were emphasized more at the whole school level.

**Table 1.** Topics of GCE in UNESCO schools

Question 13 (educators’ own teaching/management)				Question 14 (schools’ activities)			
Theme	Mean average	SD	N	Theme	Mean average	SD	N
Equality	4.1	0.7	72	Sustainable development	4.1	0.77	72
Equality between sexes	4.0	0.8	74	Equality	4.0	0.74	70
Getting acquainted with different countries and cultures	4.0	0.87	74	Getting acquainted with different countries and cultures	4.0	0.81	73
Sustainable development	4.0	0.81	73	International cooperation	4.0	0.89	71
Democracy	3.9	0.9	74	Climate change	3.9	0.74	72
Human rights	3.7	1.0	74	Human rights	3.5	0.84	72
Ecosocial understanding	3.3	0.86	74	Ecosocial understanding	3.2	0.91	73
Deconstructing global inequalities	3.2	1.0	73	Economic inequality	2.9	0.76	73
Economic inequality	3.1	0.92	73	Deconstructing global inequalities	2.9	0.94	72
Development cooperation	2.8	0.9	74	Development cooperation	2.9	0.9	72
Dialogue on religion and world views	2.8	1.17	74	Dialogue on religion and world views	2.8	1.0	73
Racialization	2.5	1.14	72	Racialization	2.3	1.02	73

Question 13: To what extent do you bring up the following topics in your teaching (teachers)/in school management (principals)? Question 14: To what extent are the following topics visible in your school’s activities? Choose the number that best describes your opinion on a scale of 1–5, with 1 = not at all, 5 = to a large extent.

Topics of GCE dealt with in UNESCO schools ranged from the le most frequently mentioned topics to the least mentioned topics, including the mean average, standard deviation, and sample size.

To get a deeper understanding of the UNESCO schools, we formulated statements based on the various typologies. Respondents were asked to mark the statements that best describe the UNESCO activities in their school (see Table 2). The most frequently picked statements were about promoting ecological sustainability, international cooperation, global ethics (e.g., human rights), and multicultural awareness; less frequently selected were statements about enabling students’ active participation and dismantling unequal global power structures. This indicates that critical (Statement 18) and economic (Statement 25) GCE stances receive less attention than moral (statement 21), cultural (statement 15), and environmental (statement 19) aspects (Oxley & Morris, 2013).

**Table 2.** UNESCO School activities

Statement	Frequency
19. Our educational institution promotes an ecologically sustainable world.	64
18. Our educational institution engages regularly in international cooperation.	63
21. Our educational institution promotes an understanding of global ethics (e.g., children’s rights, human rights).	58
15. Our educational institution promotes multicultural awareness and cultural sensitivity.	53
17. Our educational institution promotes the skills needed to operate in the international labor market and in a variety of jobs.	50
20. Our educational institution promotes an understanding of the world’s religions.	48
24. Only individual active members participate in UNESCO activities.	31
16. Our educational institution promotes an understanding of the operation of international trade and production chains and the ability to assess their fairness.	25
25. Our educational institution has developed pedagogical practices that enable the active participation of students in UNESCO activities.	24
18. Our educational institution questions and provides the tools to dismantle unequal global power structures.	18
29. Global education is part of compulsory studies for all students.	14

Question 17: From the options below, which describe the UNESCO activities in your school? Choose all applicable statements.

UNESCO school activities described by frequencies of “yes” answers to statements.

### What kinds of improvements are needed in GCE?

We were also interested in exploring how GCE could be improved in schools. Student-centered activities and involvement received the largest number of references, with 71.2% of respondents (n=67) agreeing to some extent or strongly (see Table 3), followed by the option for increasing cooperation with external agents, such as companies, organizations, and other UNESCO schools (67.1%). More collaboration between teachers and more training for educators was third (with 62.7% agreeing to some extent or strongly). The least favorite option was strengthening national steering, with 52.2% of respondents disagreeing to some extent or strongly with the statement. This is noteworthy when compared to research findings that Finnish students’ knowledge base has been good, but confidence in societal influence is low (Mehtäläinen, Niilo-Rämä, & Nissinen, 2017) it is in accord with previous statements (Table 2) indicating a low frequency of enabling students’ participation in UNESCO activities.

**Table 3:** Perceived improvements of GCED in your school

Our school needs	Strongly or to some extent disagrees	Neutral	Strongly or to some extent agree
More student-centered activities and student involvement	12.1	16.7	71.2
More cooperation with external agents (e.g., companies, organizations, UNESCO schools)	16.4	16.4	67.1
More collaboration between teachers	10.5	26.9	62.7
More training for the school staff	14.9	22.4	62.7
Stronger national steering	52.2	31.3	16.4

n=67; Question 19: How could GCED be improved in your school? Choose the option that best describes your school’s situation

### Problematic topics: Perceived obstacles and challenges in teaching GCE

We analyzed the open-ended data of question 16: “What topics in GCED are hard for you to teach? Why?” with content analysis (see results in Table 4). Approximately 61% of all responding teachers answered this question (n=38). Some explicitly responded (6 responders) that they do not have challenging topics. These and similar topics were omitted from the analysis. The primary referenced topic was racism. Other issues that received attention were: insufficiency of expertise, lack of time due to a full curriculum, minorities and diversity, controversial topics (e.g., polarization, violent extremism, and violence), gender and sexuality, abstract topics, ecological sustainability, religion, and human rights, and development cooperation received attention.

**Table 4:** Perceived obstacles and challenges in teaching GCED

Theme	Frequency	Illustrative quote
Racism	10	“Racism. Conversation polarizes easily and it is not easy to handle the topic”.
Lack of own competence	6	“The problem is often the insufficiency of my expertise”.
Minorities and diversity	6	“It’s hard because of prejudices”.
Lack of time and full curriculum	6	“The topic must be included in the curriculum, because the biggest problem in handling the issues profoundly and from multiple perspectives is lack of time”.
Violent extremism	5	“Preventing radicalization. We have not experienced that it is relevant to our students”.
Controversial issues (violence, polarization)	5	“Violence and racial discrimination need to be discussed with students in an age-appropriate manner”.
Abstract topics	4	“In general, how to make things concrete”. “I teach languages at an elementary level. At that level, it is not possible to handle very abstract topics”.
Gender and sexuality	4	“Sexual discrimination. I don’t have any personal experience of this”. / “Issues related to gender can be quite complicated”.
Ecological sustainability and climate change	3	“I have conflicting thoughts about students getting too anxious about climate change”.
Religion	3	“On topics related to religion, I don't have enough knowledge”.
Human rights	2	“It is difficult to teach human rights challenges to young pupils”.
Development cooperation	2	“How do find cooperation partners outside Europe”.

Content analysis of thematic frequency of question 16 “What themes in GCED are hard for you to teach? Why?” (n=38), 58 references = total frequency

As the following quotes suggest, teachers may also find certain topics difficult to teach if they are considered too remote for pupils and students to understand: “Eliminating global inequality and achieving quality education globally are, from the young person’s point of view, so-called distant or difficult problems to solve”.

### Ecosocial understanding and sustainable development

Ecological aspects and sustainable development were among the most significant themes throughout the study. This can be seen in the quantitative analysis of questions 13, 14, and 17 (see Tables 1 and 2). As the results indicate, sustainable development was the fourth most frequent theme in teachers’ and educators’ own work, after the topics of equality and getting acquainted with different cultures; but at the school level, it was the most frequent theme (Table 1). Ecosocial understanding is also mentioned frequently (Table 1). The significance of sustainable development can also be seen in question 17 (Table 2), in which promoting an ecologically sustainable world was the most frequently selected option to describe UNESCO school activities. The importance of sustainable development was also emphasized in the open-ended question 11 (Which principles guide your activities as a UNESCO institution) with 47 % of respondents (n =72) mentioning sustainable development among the most determining factors.

However, the responses to open-ended question 16 (What themes in GCED are hard for you to teach?) suggest that sustainable development as a theme may not always be easy to implement and integrate into the teaching as the following responses show: “Sustainability issues have seemed difficult to integrate into my teaching. Of course, we recycle in the classroom as much as the school policies permit, and sometimes we discuss the topic”. Furthermore, teaching about sustainable development may cause internal conflicts for teachers:

“In a school system which aims to lead youth to sustainable professions and lifestyles, it is hard to teach about an ecologically and socially sustainable lifestyle with devices whose production and materials are based on exploiting nature and people. Internal conflict lurks everywhere, when as a teacher or institution you don’t factually promote those aims that the ceremonial speeches and ‘agendas’ are declaring they wish to promote”.

### Human rights knowledge

The most familiar human rights instrument (Table 5) in this data (n=67) was the Universal Declaration of Human Rights (UDHR), with 80.9% considering it to be familiar or very familiar, the second being the Convention on the Rights of the Child (CRC) (68.7%), and the third the Constitution of Finland’s statements of fundamental and human rights (49.3%). Teachers have also found the CRC to be familiar in international studies (Waldron et al., 2011). The least familiar instruments in our data were the Istanbul Convention (4.5%) and the Convention on the Rights of Persons with Disabilities (CRPD) (9%). In the responses, the CRPD had the least amount of familiarity or no familiarity at all (55.2%). One reason may be that these are the newest conventions, as the CRPD was ratified in Finland in 2016 and the Istanbul Convention in 2015. These data were collected in 2020, about four to five years after ratification.

**Table 5:** Familiarity with human rights treaties and declarations among UNESCO school staff (n=67)

Instrument	Little or no familiarity (%)	Some familiarity (%)	Familiar or very familiar (%)
Universal Declaration of Human Rights (UDHR)	2.9	16.2	80.9
Convention on the Rights of the Child (CRC)	3	28.4	68.7
Constitution of Finland (fundamental and human rights)	6	44.8	49.3
European Convention on Human Rights (ECHR)	24.2	45.5	30.3
UN Declaration of HRE and Training (UNDHRET)	29.9	46.3	23.9
Convention on the Rights of Persons with Disabilities (CRPD)	55.2	35.8	9
Istanbul Convention	35.8	41.8	4.5

Other human rights topics included related to the curricula, declarations and treaties, and the role of teachers: 30% of the respondents (n=63) did not know the difference between the human rights declaration and the treaty, 54% had not noticed the human rights-related changes in the curricula, and approximately 25% gave definitions of human rights themes in the curricula. When asked about the teacher’s role as a human rights educator in question 23, the majority (n=57) described it as important.

## Discussion

This article focuses on a general understanding of describing and implementation how GCE is profiled in UNESCO schools. Furthermore, it also gives important perspectives on curricula and international standards integration in the educational field.

Some interesting results from our data compared to the typologies of Oxley and Morris (2013, p. 306) is that the critical aspects of GCE received less attention than the ecological, social, and moral GCE. From the environmental aspect, climate change, ecological sustainability, and ecosocial understanding were included in the educators’ own work and the school culture. However, the comments revealed limitations in understanding the concept of ecosocial knowledge, which was often confused with sustainability. Whereas ecosocial understanding is close to posthumanist thinking (Pulkki et al., 2020), sustainability is seen in terms of practical actions at the school level. In addition, although sustainability includes social, economic, and cultural dimensions, along with ecological ones, in our results as well as in Finnish schools in general (Cantell et al., 2020), sustainability is often understood in a simple and practical way, such as gathering rubbish from the school environment or recycling. Deeper ecosocial and cultural elements are missing. Certainly, many teachers have a comprehensive understanding of this issue (Cantell et al., 2020). However, although the results show limitations of teachers’ understanding of ecosocial thinking, we argue that adding this concept in the curricula is an important step to strengthening posthumanist thinking, where interactions between humans and the environment are in balance. This is especially important in the Anthropocene era and the huge global environmental crisis.

Economic inequality and active formulations that deconstruct global inequality received less attention, and such topics were in some cases considered even more difficult than, for example, international cooperation, democracy, and equality as commitments. International cooperation was often referred to, strengthening the hypothesis that UNESCO principles are often realized through internationalization. These results also indicate that, for example, Andreotti’s description of critical GCE as the recognition of asymmetrical and unequal power relations is less emphasized (Andreotti, 2006, Table 1).

Difficulties were expressed particularly in relation to racism. Racialization was less handled than other issues, and in open-ended questions, racism was referenced several times (Table 4). This is not surprising, considering Finland’s high levels of racism (FRA, 2017) and its previously noticed unwillingness to handle domestic human rights problems (Toivanen, 2007, p. 43). References to difficulties in relation to racism may be interpreted in connection to fewer mentions in critical statements of e.g., deconstructing inequalities that these are challenging topics for Finnish educators.

The mention of difficulties connected to teaching human rights is similar to studies that show teachers find age-appropriate teaching to be challenging (Cassidy et al., 2014; Struthers, 2016). Results of familiarity with human rights treaties were also in alignment with, for example, Irish teachers perceiving the CRC to be the most familiar human rights treaty (Waldron et al., 2011, p. 26). Our data diverged in that the Constitution of Finland’s fundamental and human rights were less familiar than UDHR or CRC, which is a bit surprising. In comparison to Ireland, the Constitution of Ireland was the most familiar (ibid.). Interesting in this result is that the constitution is strong legal instrument and UDHR is not legally binding, although it is historically relevant. This supports the claim that human rights may be taught as part of history rather than addressing their judicial role in society, supporting the previous research results that HRE in Finland is detached from a judicial norm base (HRC, 2014; Matilainen, 2011; Kasa et al., 2021).

One interesting aspect of our data originated from UNESCO educators’ reporting how they have noticed curricula changes related to human rights: 54% (n=63) responded that they have not paid attention to them. This indicates that although the curricula are explicit, this does not mean that educators necessarily take them into account. Moreover, there is a conflict in this data, stating that promoting global ethics (including human rights, for example) was often referred to as a UNESCO activity (Table 2), but at the same time the specific questions indicate a lack of knowledge (Table 5). This result was also seen by Shultz et al. (2009), who concluded that few educators in UNESCO ASPnet schools considered themselves educators in matters related to human rights. In our data, the majority perceived the teacher’s role as a human rights educator to be important.

In conclusion, from the specific focus on ecosocial understanding and human rights, our results indicate that if it is assumed curricula changes are sufficient to change educators’ knowledge, in this case, it seems, curricular emphases do not transform into knowledge without efforts at continuous learning. Many professionals—even in the supposedly more “active” schools—do not necessarily even notice these changes. This finding is in line with earlier research, which shows that school reforms, including curricular reforms, seldom successfully transform classroom practices without careful focus on the implementation of the reforms, as well as on teachers’ central involvement in envisioning the intended changes (Pietarinen et al., 2017; Sarason, 1990). With regard to improving GCE in schools, similar ideas and suggestions emerged in our study as stated by Shultz et al. (2009). Professional development for teachers and engaging students on a more profound level were mentioned in both studies, with our study also highlighting a need for more collaboration between teachers and external agents outside schools.

## Conclusions

Our study suggests that some improvements can be made, specifically at UNESCO schools, to foster and advance GCE. Among those mentioned were more student-centered approaches to GCE, collaboration between teachers, in-service education, and collaboration with external agents outside school. These pedagogical perspectives are very central in current textbooks on teaching and learning (e.g., Cantell & Kallioniemi, 2016). Collaboration and working together are also emphasized in textbooks on pedagogical leadership (Holappa et al., 2021).

When analyzing the results that emerged from our data through the typologies of GCE (Andreotti, 2006; Oxley & Morris, 2013), economic, critical, and spiritual GCE received less attention. Fewer references to, for example, spiritual GCE are connected to international research (Yemini et al., 2019). Difficulties were perceived in relation to racism and racialization, which is not surprising in the context of high racism rates (FRA, 2017) and the number of hate crimes (Rauta, 2017) in Finland.

Regarding the curricula and understanding of educators, we conclude that on closer examination, both ecosocial understanding and human rights are comprehended differently than the curricula suggest. We propose that when complex issues are introduced into curricula, sufficient support from the side of educational policy should be guaranteed to teachers.

We acknowledge that our study focuses only on teachers’ and principals’ perspectives. To arrive at a deeper understanding of how the role of GCE is understood in schools, it would be highly important to also study pupils’ and students’ views on the matter. A comparison between UNESCO ASPnet schools and other educational institutions of views regarding GCE would also provide a more versatile view of the matter.
